# Comparison of Genomic Driver Oncogenes in Vietnamese Patients With Non–Small-Cell Lung Cancer in the United States and Vietnam

**DOI:** 10.1200/JGO.18.00086

**Published:** 2018-11-13

**Authors:** Kim-Son H. Nguyen, Henning Stehr, Li Zhou, Anh-Hoa Nguyen, Pham Nhu Hiep, Nguyen Van Cau, Phan Canh Duy, Richard Thorp, Heather A. Wakelee, Maximilian Diehn, Joel W. Neal

**Affiliations:** **Kim-Son H. Nguyen**, Palo Alto Medical Foundation, Mountain View; **Henning Stehr**, **Li Zhou**, **Heather A. Wakelee**, **Maximilian Diehn**, and **Joel W. Neal**, Stanford University School of Medicine, Stanford; **Anh-Hoa Nguyen**, AK Research Group, Redwood City, CA; **Pham Nhu Hiep**, **Nguyen Van Cau**, Hue Central Hospital; **Phan Canh Duy**, Hue University School of Medicine and Pharmacy, Hue, Vietnam; and **Richard Thorp**, BioreclamationIVT, Chestertown, MD.

## Abstract

**Purpose:**

Discoveries of oncogenic driver alterations in non–small-cell lung cancer (NSCLC) have been accompanied by the development of effective targeted therapies. The frequencies of these mutations vary between populations but are less well characterized in the Vietnamese population. In this study, we analyzed the frequencies of lung cancer driver oncogenic alterations in Vietnamese patients compared with Vietnamese patients treated in the United States.

**Methods:**

We collected data on tumor and disease characteristics of Vietnamese patients with NSCLC treated at Stanford. In addition, we collected NSCLC tumor specimens from patients with NSCLC diagnosed in Hue, Vietnam, and performed next-generation–based genotyping on these samples. The molecular and clinical characteristics of these groups were compared.

**Results:**

Fifty-nine Vietnamese patients were identified at Stanford. Of the 44 patients with molecular testing results, there were 21 (47.7%) with *EGFR* alterations, six (13.6%) with *ALK* alterations, two (4.5%) with *KRAS* alterations, one (2.3%) with *BRAF* alterations, and no *ROS1* or *RET* alterations. Across all stages, the median overall survival for patients with a tumor having a targetable genomic alteration driver mutation was 42.4 months, compared with 27.1 months for patients without such alterations. In the 45 genotyped samples from Vietnam, there were 26 (57.8%) with *EGFR*, 11 (24.4%) with *KRAS*, and one each (2.2%) with *ALK*, *ROS1*, and *RET*.

**Conclusion:**

The majority of tumors from both Stanford and Vietnam had targetable oncogenic alterations. This suggests that routine implementation of molecular testing may have a significant, positive impact on the treatment of Vietnamese patients with NSCLC, but affordability of testing and treatments remains a barrier to adoption.

## INTRODUCTION

Discoveries of oncogenic mutations in non–small-cell lung cancer (NSCLC) over the past decade have led to significant treatment advances with orally available targeted therapies. Tumors driven by activating *EGFR* mutations, commonly including exon 19 deletions or the L858R point mutation on exon 21, have been shown in multiple randomized controlled trials to be more sensitive to EGFR tyrosine kinase inhibitors (TKIs) than to cytotoxic chemotherapy in the first-line setting.^1-3^ Similarly, tumors with *ALK* gene rearrangements are also highly sensitive to ALK TKIs, such as crizotinib, ceritinib, and alectinib.^4-6^ Another druggable target in NSCLC is the *ROS1* gene rearrangement, and ROS1-positive tumors are also highly sensitive to crizotinib.^7^ Unfortunately, despite being more prevalent than other genomic alterations, *KRAS*-mutant tumors seem to have little sensitivity to targeted therapies.

The frequencies of these oncogenic alterations in NSCLC seem to be widely different among geographic populations for unclear reasons. *EGFR* mutations are known to occur in approximately 15% of NSCLCs in the Western population, yet closer to 50% in the East Asian population.^1,2,8,9^ *ALK* gene rearrangements are consistently observed in approximately 4% of NSCLC tumors in Western and East Asian populations^10-17^ and *ROS1* gene rearrangements in approximately 1% to 2% of NSCLC in white and Chinese populations.^18-20^ Because most of the clinical trials on targeted therapies for NSCLC have been conducted in the United States, Europe, China, Japan, South Korea, and Taiwan, less is known about the frequencies of driver mutations in NSCLC in developing East Asian countries. In particular, molecular profiles of NSCLC remain relatively uncharacterized in Vietnam, a World Bank–designated lower-middle–income country with more than 90 million people. Vietnam is struggling to contain a rapidly growing cancer burden, where lung cancer ranks second only to liver cancer in incidence and mortality, with more than 20,000 patients reported per year. Molecular analysis is rarely performed in Vietnam because of high costs and limited availability. This article describes the molecular profiles of NSCLC in Vietnamese patients by comparing two cohorts of Vietnamese patients with lung cancer: those diagnosed in Stanford, CA, and those diagnosed in Hue, Vietnam, using modern diagnostic assays.

## METHODS

### Study Design

This was a retrospective study of oncogenic alterations in patients of Vietnamese origin with NSCLC of all histologies. Under an institutional review board–approved protocol, the Stanford Cancer Institute Research Database was used to identify patients with NSCLC of Vietnamese origin on the basis of self-reported ethnicity, language, or country of origin. Patients who were seen for an initial visit to the Stanford Cancer Center thoracic oncology clinic between April 2009 and December 2013 were included. We collected data on sex, smoking status, smoking pack-years, date of pathologic diagnosis, date of first clinic visit, date of last follow-up, date of death, tumor histology, disease stage, molecular data, and treatment data (including modality of treatment, start date, and date of progression). The data cutoff date was May 1, 2014. Another 46 samples of NSCLC tumors were obtained from patients who underwent surgical resection of tumors between December 2012 and February 2014 at Hue Central Hospital, Hue, Vietnam, and were collected under an institutionally approved protocol that also allowed research testing of clinical specimens outside the country. The majority of these patients had the associated clinical variables: sex, smoking status, tumor histology, and disease stage. Treatment and clinical outcome data were not available.

Molecular testing was performed as part of routine clinical care of the Stanford patients as follows: ALK status was determined using the standard break-apart *ALK* fluorescent in situ hybridization assay^13^; ROS1 status was determined with break-apart fluorescent in situ hybridization^18^; and *EGFR*, *KRAS*, and other cancer-related genes were determined using DNA sequencing (2007 to 2011) or SNaPshot (2011 to 2013).^21^ Molecular testing was performed in the Hue specimens using a research version of the next-generation sequencing–based Stanford Actionable Mutation Panel for solid tumors, which, subsequent to this study, became the routine next-generation sequencing–based testing platform for clinical use at Stanford.^22^

### Statistical Analyses

Descriptive statistics were performed for oncogenic alteration frequencies, sex, smoking status, histology, and disease stage. Frequencies of oncogenic alterations were compared among different subgroups with χ^2^ or Fisher's exact test. Survival analyses for the Stanford population were performed using the Kaplan-Meier method. All statistical analyses were programmed using SAS 9.2 statistical software (SAS Institute, Cary, NC). Statistical significance was assumed for a two-tailed *P* value less than .05.

## RESULTS

### Patient Characteristics

In the Stanford cohort, 59 patients of Vietnamese origin were identified, including 29 men (49.2%) and 30 women (50.8%), with a mean age at diagnosis of 63.2 years, ranging from 34 to 85 years. In the Hue cohort, 24 patients (52.2%) were male, 15 (32.6%) were female, and no sex information was available for seven patients (15.2%); the mean age was 57.9 years, ranging from 38 to 75 years (Table 1).

**Table 1:**
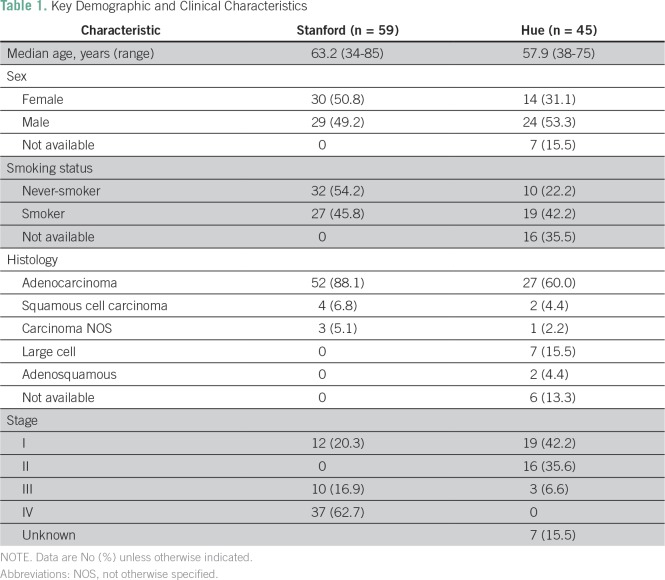
Key Demographic and Clinical Characteristics

In the Stanford group, 32 (54.2%) were never-smokers, 26 (44.1%) were former smokers, and only one (1.7%) was a current smoker. Twenty-eight of the 30 women (93.3%) were never-smokers, whereas only four of the 29 men (13.8%) were never-smokers. The median number of cigarette pack-years for smokers was 24. In the Hue cohort, 10 patients (21.8%) were never-smokers, 19 (41.3%) were smokers, and 17 (37%) patients had no information collected on smoking status; pack-year information was not collected.

### Tumor Characteristics and Molecular Profiles

The majority of the tumors from the Stanford cohort, 52 of 59 (88.1%), were adenocarcinomas, with only four (6.8%) squamous cell carcinomas and three (5.1%) carcinomas not otherwise specified. Interestingly, the histologies from Hue were different, with 27 (58.7%) adenocarcinomas, two (6.3%) squamous cell carcinomas, one (2.2%) carcinoma not otherwise specified, seven (15.2%) large cell carcinomas, and two (6.3%) adenosquamous carcinomas. In seven patients (15.2%), no specific histology was given (Table 1).

At Stanford, the majority of patients presented with an advanced stage: 12 patients (20.3%) at stage I, none at stage II, 10 (16.9%) at stage III, and 37 (62.7%) at stage IV. In contrast, most of the samples collected at Hue were from patients presenting with a local stage: 39 (84.8)% at stage I, 19 (41.3%) at stage II, three (6.5%) at stage III, and no patients with stage IV.

**Fig 1:**
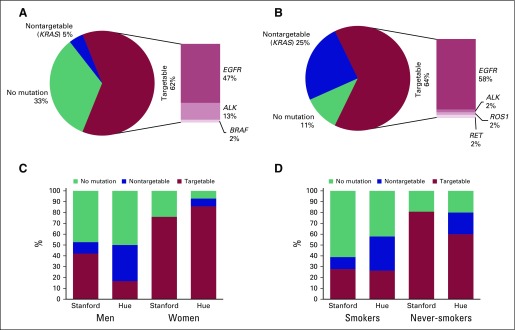
Oncogenic alterations in Vietnamese patients with non–small-cell lung cancer. Frequency of oncogenic alterations for (A) the overall Stanford cohort, (B) the overall Vietnam cohort, (C) each cohort subdivided by sex, and (D) each cohort by smoking status. ALK, anaplastic lymphoma kinase; EGFR, epidermal growth factor receptor; ROS1, repressor of silencing 1.

In the Stanford cohort, 44 of the 59 patients underwent molecular testing. Of the tumors that were tested, 47.7% had an EGFR-activating mutation, 13.6% had an *ALK* gene rearrangement, and 4.5% had a *KRAS* mutation. One third (33%) had no identified oncogenic alteration (Fig 1A). Of the 21 patients with *EGFR*-mutant NSCLC, 17 (81.0%) were never-smokers, and 16 (76.2%) were female. Of the six patients with an *ALK* gene rearrangement, three were female, and three never smoked. Interestingly, one of the ALK-positive tumors had a confirmed squamous cell carcinoma. No *ROS1* gene rearrangements were found among 11 patients tested. One patient (2.3%) was found to have a *BRAF* mutation. Overall, 21 of 32 never-smokers (65.6%) and seven of 27 smokers (25.9%) had a targetable driver mutation (*EGFR*, *ALK*, *BRAF*). Of note, we excluded *KRAS* mutations from the list of targetable driver mutations because there was no standard targeted therapy for KRAS-mutant NSCLC.

In the Hue cohort, all 45 patients had molecular testing. Twenty-six tumors (57.8%) had an *EGFR*-activating mutation, and 11 tumors (24.4%) had a *KRAS* mutation (Fig 1B). There was one patient (2.2%) each with *ALK* and *ROS1* gene rearrangement and one patient (2.2%) with RET translocation.

When comparing the patients by site and gender, more female patients had driver oncogenic alterations than males (Fig 1C), and more never-smokers had driver alterations than smokers (Fig 1C). Also, within gender and smoking subgroups, Hue patients consistently had a higher frequency of driver alterations than Stanford patients.

### Clinical Outcomes

**Fig 2:**
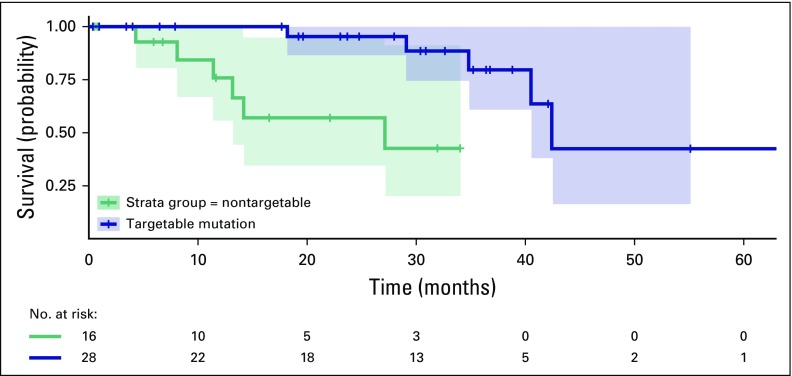
Overall survival of Vietnamese patients with non–small-cell lung cancer treated at Stanford. Kaplan-Meier curve of overall survival of the Stanford cohort, divided by patients with a targetable mutation, versus patients with a nontargetable mutation or no identified mutation. ALK, anaplastic lymphoma kinase; EGFR, epidermal growth factor receptor.

Treatment and clinical outcome data were only available for the Stanford patients. Of the 59 patients in this cohort, 12 had died at the time of data cutoff. The median overall survival (OS) for all patients was 40.5 months (95% CI, 29.1 to 68.1 months). The estimated median OS for patients who were tested and found to have no targetable driver mutation was 27.1 months (95% CI, 8.1 months to not reached), and that for patients with a targetable driver mutation was 42.4 months (95% CI, 34.8 to 68.1 months log rank *P* = .00154; Fig 2).

## DISCUSSION

Our study was an international comparison of the patterns of oncogenic alterations in patients with NSCLC of Vietnamese origin treated in the United States and in Vietnam. There is a paucity of research on lung cancer in the Vietnamese population living in Vietnam and overseas. A retrospective study of 1,124 Asian American patients with NSCLC from 2001 to 2005 in Southern California included 369 Vietnamese Americans, of whom 39.7% were never-smokers. The median age of diagnosis for Vietnamese never-smokers was 65 years, and that for smokers was 67 years. Men made up approximately 28% of the never-smokers and 85% of the smokers with lung cancer. Adenocarcinomas comprised approximately half of the Vietnamese NSCLC histologies, and more than half of the Vietnamese patients were diagnosed at stage IV. In this study, the authors found no statistically significant difference in OS among five Asian American subgroups.^23^ Another study of Asian American patients with cancer in California revealed that Vietnamese men and women had the highest incidence of lung cancer among the five Asian American groups: Chinese, Filipino, Japanese, Korean, and Vietnamese. Vietnamese patients were found to have the lowest income and education level among the Asian ethnic groups.^24^ There exist some published data comparing cancer incidence rates in the overseas Vietnamese population to Vietnamese living inside Vietnam. In one study, the lung cancer incidence rate was 29.8 per 100,000 in Hanoi, Vietnam, compared with 55.9 per 100,000 in Vietnamese patients from California and SEER databases.^25^ The authors argued that the difference might have been due to environmental differences or underdiagnosis and underascertainment in Vietnam.

We found that 63.6% of Vietnamese patients at Stanford and 64.4% of those in Hue, Vietnam, had an oncogenic alteration. We confirmed a clinical association between never-smoking status and the female sex with having an *EGFR*-activating mutation. The first published literature on the molecular profiles of Vietnamese patients with lung cancer was the PIONEER study, a prospective study examining *EGFR* mutations in Asian patients with advanced pulmonary adenocarcinoma.^26^ This study included 121 patients from a hospital in northern Vietnam. The frequency of *EGFR*-activating mutations was 64.2% in the Vietnamese cohort from this study. Another study from the University of Medicine and Pharmacy in Ho Chi Minh City, Vietnam, performed Sanger sequencing on 135 samples and found *EGFR* mutations in 40.7%.^27^ In August 2015, researchers from Bach Mai Hospital, Hanoi, Vietnam, presented an abstract at a national oncology conference showing that of 166 tested NSCLC samples at their hospital, 38.0% had *EGFR*-activation mutations. These studies together with our study showing that 57.8% of patients from Hue, Vietnam, had *EGFR* mutations and 47.7% of Vietnamese patients at Stanford had *EGFR* mutations, show that *EGFR* mutations are frequent in this population. There do not seem to be published data on the rate of other driver oncogenes in Vietnamese patients. Our Stanford population also shows a higher-than-expected ALK-positive frequency of 13.6%, but with a limited sample size, this may be due to statistical variation. The single instances of ALK, ROS1, and RET identified in the cohort from Vietnam are consistent with our expectation that these are low-frequency, but present, alterations in this population. For the patients treated at Stanford, OS was superior in those who had a targetable oncogenic alteration, as demonstrated in other populations.^28^

This study has some limitations. For both the Stanford and Hue cohorts, the sample size was relatively small. All patients in the study received care at academic centers; thus, the findings may not be generalizable to the broader Vietnamese population. At Stanford, there might have been patients of Vietnamese origin who were not included in the study if they did not have a Vietnamese name or if they declined to declare their ethnicity or language. In addition, the stages at presentation varied widely between the two cohorts. At Stanford, as expected, a majority of patients presented at stage IV. However, almost all of the samples obtained from Hue were from patients presenting at stage I or II, with only a few at stage III and none at stage IV, because the samples suitable for collection and sharing for research, including molecular analysis, were generally surgical samples, and patients at an advanced stage are unlikely to undergo surgery. However, there may be understaging in Vietnam, with limited access to the use of full-body positron emission tomography/computed tomography scanners and contrast-enhanced head magnetic resonance imaging; therefore, it is likely that a subgroup of patients in the Vietnamese cohort had more advanced disease. Furthermore, in Vietnam, advanced-stage lung cancer may not be pathologically diagnosed, because it is not uncommon for patients who present with radiographically presumed advanced-stage lung cancer to forgo a diagnostic biopsy and proceed directly to palliative treatment. Another weakness of the study results from not performing a single uniform molecular test on all patients. For example, of the 59 total patients at Stanford, only tumors from 44 patients had any molecular testing, and some patients only had EGFR testing, whereas others had a more comprehensive multiplex mutation panel. This is because of the improvements in clinical molecular testing over time in NSCLC, but samples did not remain on the majority of patients to permit performance of next-generation sequencing. Therefore, it is difficult to accurately assess the absolute frequencies of various oncogenic alterations in this cohort. Last, survival data are unavailable on the patients from Hue, preventing a comparison between the sites or assessment of survival by molecular alteration subgroups.

Despite these limitations, this study revealed that a significant majority of patients with lung cancer of Vietnamese origin have an oncogenic alteration that can be targeted therapeutically. This is consistent with reports from other East Asian countries in which the rates of *EGFR* mutations are quite high. Although additional studies are needed to confirm these interesting findings, this study underscores the need for increased access to affordable molecular testing and targeted therapies. Unfortunately, in a country where the per capita median income is US $1,130 per year, an *EGFR* mutation test costs between US $1,000 and $2,000 (Q.T. Khanh, personal communication, October 2012). Furthermore, the majority of patients cannot afford the approximate cost of US $1,000 per month or more for an EGFR TKI (N.V. Cau, personal communication, August 2015). The disparity in lung cancer treatment, and cancer care in general, is apparent for those of us who have cared for patients in both countries. Personalized lung cancer treatment with targeted therapies, which could only become widespread with lower-cost molecular testing and affordable oral TKIs, remains a worthwhile but elusive goal in Vietnam.
